# Beneficial Effects Exerted by Paeonol in the Management of Atherosclerosis

**DOI:** 10.1155/2018/1098617

**Published:** 2018-11-07

**Authors:** Li Lu, Yating Qin, Chen Chen, Xiaomei Guo

**Affiliations:** Department of Cardiology, Tongji Hospital, Tongji Medical College, Huazhong University of Science and Technology, Wuhan 430030, China

## Abstract

Atherosclerosis, a chronic luminal stenosis disorder occurred in large and medium arteries, is the principle pathological basis of cardiovascular diseases with the highest morbidity and mortality worldwide. In oriental countries, traditional Chinese medicine Cortex Moutan has been widely used for the treatment of atherosclerosis-related illnesses for thousands of years. Paeonol, a bioactive monomer extracted from Cortex Moutan, is an important pharmacological component responsible for the antiatherosclerotic effects. Numerous lines of findings have established that paeonol offers beneficial roles against the initiation and progression of atherosclerotic lesions through inhibiting proatherogenic processes, such as endothelium damage, chronic inflammation, disturbance of lipid metabolism, uncontrolled oxidative stress, excessive growth, and mobilization of vascular smooth muscle cells as well as abnormality of platelet activation. Investigations identifying the atheroprotective effects of paeonol present substantial evidence for potential clinical application of paeonol as a therapeutic agent in atherosclerosis management. In this review, we summarize the antiatherosclerotic actions by which paeonol suppresses atherogenesis and provide newly insights into its atheroprotective mechanisms and the future clinical practice.

## 1. Introduction

Exposed to numerous social and health problems such as aging of population, progressive urbanization, elevated energy intake, reduced physical exercise, and air pollution, more and more people are insulted by cardiovascular diseases (CVDs) [1]. According to the statistics of the World Health Organization, more than 40% of deaths of noncommunicable diseases are attributable to CVDs annually [2]. Atherosclerosis, characterized by multifactor-induced vascular stenosis occurred in large- and medium-sized arteries, is a crucial predisposed pathogenic process toward CVDs [3]. It has been demonstrated that statins are effectively used for the treatment of atherosclerosis. However, 5%–20% of patients with indications for statin therapy show inability to tolerate routine dosages due to muscle symptoms caused by statins [4].

Cortex Moutan, the root bark of *Paeonia suffruticosa* Andrews, has been widely applied as a traditional Chinese medicine (TCM) in the prevention and management of various diseases for thousands of years, such as CVD, diabetes, arthritis, and cancer [5]. Paeonol (2′-hydroxy-4′-methoxyacetophenone) is a bioactive constituent extracted from Cortex Moutan and has been reported to possess extensive pharmacological properties for alleviating atherosclerotic lesions, which is associated with improvement of endothelial injury, repression of vascular smooth muscle cell (VSMC) proliferation and migration, amelioration of inflammation and oxidative stress, inhibition of platelet activation and aggregation and decrease of blood lipids, etc. [6–11]. In oriental countries, paeonol has been employed alone or in combination with other TCMs to effectively protect the cardiovascular system, suggesting that paeonol is potentially to act as an alternative or complementary agent for compensating for the limited efficiency and uncertain safety of modern drugs regarding atherosclerosis treatment [4, 12–14]. Considering the pharmacological activities and therapeutic potentials of paeonol in dealing with atherosclerosis, we put forward an overview concerning the atheroprotective roles of paeonol and the underlying mechanisms identified in preclinical studies.

## 2. Pharmacological Features of Paeonol

### 2.1. Bioactive Components in Cortex Moutan

**Figure 1:**
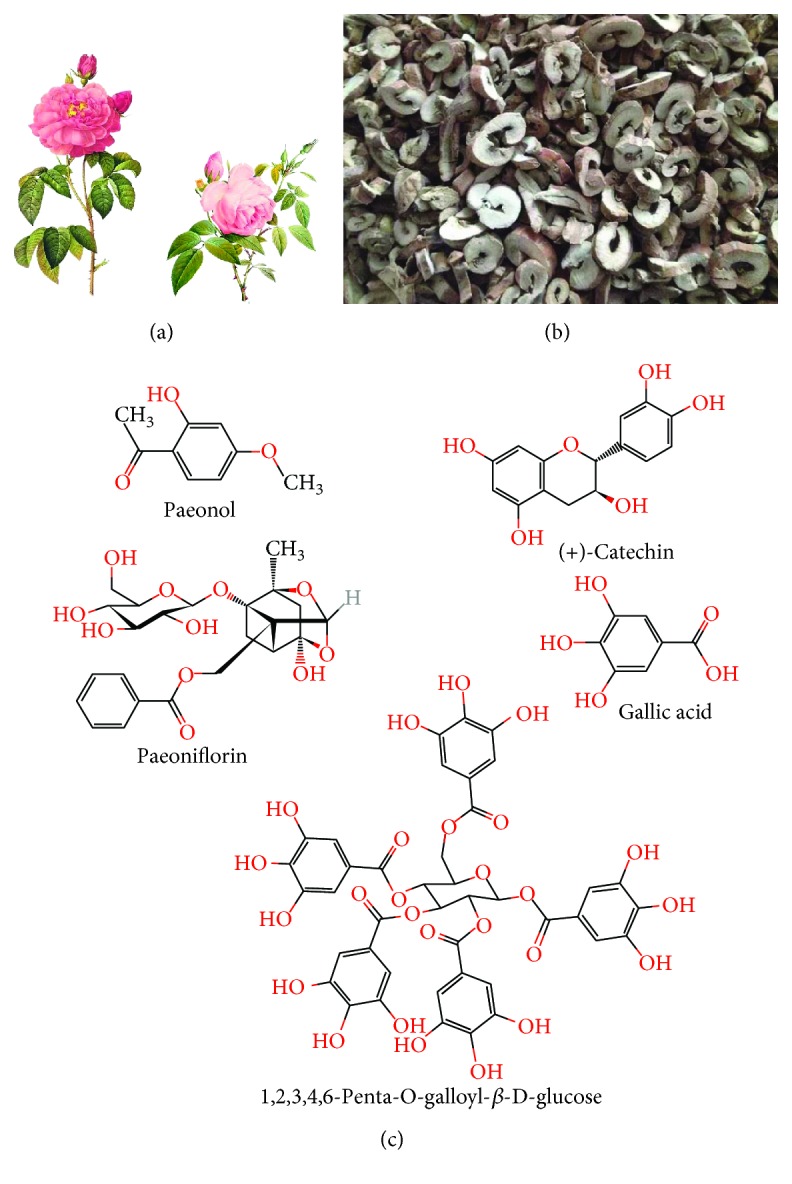
The whole plant and root bark of *Paeonia suffruticosa* Andrews and relevant isolated components. (a) *Paeonia suffruticosa* Andrews is a kind of elegant ornamental plant with great medicinal value. (b) Cortex Moutan, the root bark of *Paeonia suffruticosa* Andrews, contains a variety of bioactive pharmacological compounds including paeonol. (c) The chemical structural formula of the main components extracted from Cortex Moutan.

Since multiple lines of evidence have clarified the cardioprotective effects of Cortex Moutan, the molecular mechanisms are difficult to be recognized and accepted because of its complex mixture nature. Increasing attention paid by the cardiovascular research community is focused on the bioactive chemical monomers comprised in Cortex Moutan responsible for the pharmacological abilities [5, 15]. Phytochemical studies indicate that there are more than 80 compounds isolated from Cortex Moutan, mainly divided into the following categories with different structural formulas: monoterpenoid glycosides, flavonoids, tannins, phenols, and paeonols. Among them, paeoniflorin, catechin, 1,2,3,4,6-penta-O-galloyl-*β*-D-glucose, gallic acid, and paeonol are the representative extracts in the above groups, respectively (Figure 1) [15, 16]. In terms of the involvement of these chemicals in atherosclerosis development, paeonol is the main bioactive component which is extensively investigated.

### 2.2. Pharmacokinetics of Paeonol

It is universally established that pharmacokinetic detection is beneficial to evaluate the efficacy and possible toxicity of herbal products and explore the interactions between medicinal herbs among the preparations. With some advanced detection methods, the pharmacokinetic researches of paeonol have been broadly performed in the past years (Table 1) [17–21]. Paeonol is rapidly absorbed into the circulation from the intestinal tract after oral administration and quickly distributed in multiple organs including the heart, liver, kidney, and brain without long-term accumulation, as explained by short *T*_max_ and *T*_1/2_ [17, 18]. This rapid clearance of paeonol from the body appears to guarantee its safety. Furthermore, the level and duration of paeonol in the heart and brain could be significantly increased via coadministration with danshensu, which might be helpful to interpret the synergistic actions of combination remedy amalgamating paeonol and danshensu in treating cardiovascular disorders [13, 18, 22]. However, fast and complete first-pass metabolism of paeonol, along with the features of low aqueous solubility and high volatility, determines its poor bioavailability.

### 2.3. Drug Delivery System

A number of drug delivery systems connected with paeonol have been developed to enhance the dissolution rate and unsatisfactory bioavailability, because the hydrophobicity of paeonol hinders its clinical application as a promising therapeutic agent [23–25]. Microemulsion gel, transethosome, porous microsphere, liquid crystalline nanoparticle, and microsponge formulations have been designed for transdermal delivery of paeonol. Results show that these carriers, with high encapsulation efficiency and stability, are biocompatible with paeonol and dramatically raise skin permeability, control release of drug, extend time of drug residence in local tissues, and lower irritation to covered areas, implying superior effects of these complexes in treating skin diseases [23, 26–29]. Additionally, paeonol-loaded nanoparticles are more effective in cancer treatment when compared to paeonol alone [30]. Nonetheless, there is no paeonol-related drug delivery system which has been designed and prepared for atherosclerosis therapy up to now. Given that cancer and atherosclerosis share some common pathogenic mechanisms, vehicles loading paeonol used for cancer management might be suitable for alleviating atherosclerosis progression, which is needed to be further elucidated [31]. Moreover, nanoemulsions prepared by Chen et al. are likely to strengthen therapeutic effects of paeonol in the cardiovascular system, for which augment the bioavailability of paeonol by enhancing its oral absorption and transport in the digestive tract through blocking p-glycoprotein regulated efflux [32]. In addition, stents carrying paeonol-laden microparticles or poly (butyl-2-cyanoacrylate) nanocapsules are likely to have high efficiency in preventing restenosis and stent thrombosis occurrence after percutaneous coronary intervention, by the fact that the formulations could slow and control the release of paeonol, which probably sustainedly inhibited VSMC proliferation and platelet activation in the local environment [23, 24].

## 3. Mechanism of Action Underlying Paeonol Alleviates Atherosclerosis

**Figure 2:**
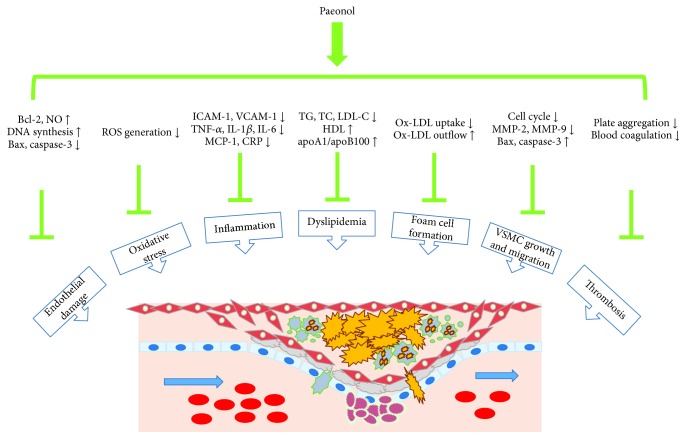
The antiatherosclerotic effects by which paeonol alleviates the development of AS.

Accumulating studies support the notion that atherosclerosis is a multifaceted vascular impairment involving functional abnormalities of diverse cell types like endothelial cells (ECs), macrophages, VSMCs, and platelets. Endothelium damage, chronic inflammation, disturbance of lipid metabolism, uncontrolled oxidative stress, excessive growth, and mobilization of VSMCs and abnormality of platelet activation are important contributors to atherogenesis [33, 34]. Targeting these proatherogenic processes is indicated to be the pivotal mechanisms underlying paeonol mitigates atherosclerotic lesions and subsequent cardiovascular events (Figure 2).

### 3.1. Amelioration of Endothelial Injury

The intact vascular endothelium, known as nature's container for circulating blood in vivo, has been delineated to be deeply associated with diverse biological processes. When incited by proatherogenic factors, apoptotic signaling in ECs is amplified and the barrier of arterial vasculature is deranged, which causes increased permeability of the endothelial lining of lesion-prone areas, followed by trapping and epigenetic modification of blood lipoproteins as well as macrophage deposition and succedent foam cell formation in the subendothelial region, thereby favoring atherosclerosis initiation [33]. Independent research teams have uncovered that paeonol could improve endothelial damage by enhancing endothelial nitric oxide synthase- (eNOS-) induced production of nitric oxide (NO) in ECs in response to diverse stimuli, owing that NO is an EC-protecting factor capable of repressing activities of apoptosis-related pathways and elevating cellular survival rates [9, 35–37]. Through suppression activation of phosphatidylinositol 3-kinbase (PI3K)/Akt/nuclear factor kappa B (NF-*κ*B) and lectin-like low-density lipoprotein receptor-1 (LOX-1)/p38/NF-*κ*B cascade, paeonol inhibits apoptosis and increases viability of EC damaged by lipopolysaccharides (LPS) and ox-LDL, as seen by downregulation of caspase-3 level and LDH leakage and upmodulation of Bcl-2 expression and OD value of MTT test [38, 39]. Other aspects associated with endothelium dysfunction encompass premature senescence, aberrant autophagy and microRNA (miRNA) mediation, reactive oxygen species (ROS), and inflammation stimulation [33]. As untimely aging of ECs deteriorates their actions of growth and antioxidation, pretreatment with paeonol is showed to reduce the number of senescent cells, propel cell cycle, and DNA synthesis and then restore abilities resistant to dysfunction in the model of endothelial senescence, which is linked to mediation of sirtuin 1 (Sirt1)/p53 axis [40, 41]. Emerging findings depict that autophagy is an evolutionarily conserved process degrading own damaged proteins and macromolecule substances and uncontrolled autophagy results in atherosclerosis-related vascular ECs death [42]. Paeonol has been demonstrated to ameliorate cell injury induced by excessive autophagy in ox-LDL-triggered ECs through raising the level of miR-30a which downmodulates expression of Beclin-1 and LC3II [6]. Moreover, another mechanism by which paeonol recovers the proliferation of ECs damaged by ox-LDL is ascribe to paeonol-induced decrement of proapoptotic miR-21 expression and following TNF-*α* release [43]. Taken together, it is rational to discern that paeonol possesses great beneficial potentials in the treatment of endothelium dysfunction.

### 3.2. Inhibition of Oxidative Stress

Under physiological conditions, generation and elimination of ROS is in a dynamic equilibrium. In diseases, the overproduction of oxidants or shortage of antioxidants leads to the imbalance of the redox status, causing ROS overload and then proximal and distal impairments called oxidative stress. Considerable documentations reveal that oxidative stress exerts positive roles in the pathogenesis of atherosclerosis [37, 44]. Experimental data have manifested that paeonol obviously lowers ROS content, abrogates the upregulation of MDA and oxidized low-density lipoprotein (ox-LDL), restores the level of Bcl-2/Bax and caspase-3, and decreases expression of tumor necrosis factor (TNF)-*α*, interleukin (IL)-1*β*, IL-6, and monocyte chemotactic protein (MCP)-1 in the oxidative stress environment [45–47]. These findings show that encumbering oxidative stress-evoked acceleration of lipid peroxidation, induction of vascular endothelial injury, and activation of inflammatory pathways are imperative components in the antiatherogenic effects of paeonol. Investigations on the molecular mechanisms suggest that paeonol could induce ROS decline through activating AMP-activated protein kinase *α* (AMPK*α*)/peroxisome proliferator-activated receptor *δ* (PPAR*δ*) cascade and blocking endoplasmic reticulum (ER) stress signaling, followed by reduction of NADPH oxidase (NOX) which is the main enzyme catalyzing ROS generation, indicating that synthesis inhibition of ROS is an important action of paeonol to improve oxidative stress [9, 44, 48]. In terms of the effects of paeonol on the antioxidative system, previous evidences had uncovered that paeonol was capable of increasing contents of antioxidants and scavenging ROS-evoked cardiac and cerebral injury via inducing nuclear factor E2-related factor 2 signaling and downstream expression of heme oxygenase-1, superoxide dismutase, catalase, and glutathione peroxidase [13, 49]. Then, the potent abilities of elevating antioxidant enzymes of paeonol might directly eliminate ROS and then ameliorate oxidative stress-elicited vascular wall damage.

### 3.3. Mitigation of Inflammatory Response

Tremendous basic studies have elaborated the essential atheroprone impacts of inflammation in all stages of atherosclerosis from fatty streak formation to luminal occlusion. Activation of inflammatory cascades in vascular ECs stimulates the biosynthesis of adhesion molecules (vascular cell adhesion molecule-1 (VCAM-1), intercellular adhesion molecule-1 (ICAM-1), and E-selection) and chemokines (MCP-1) which promote recruitment and retention of circulating monocytes in the intima where they differentiate into macrophages and aggravate atherosclerotic lesions via changing into foam cells and secreting vast proinflammatory factors [3, 50]. In vitro experiments confirm that paeonol forcefully retards the detainment of monocytes by ECs via weakening the expression of VCAM-1 and ICAM-1 in ECs upon stimulation of TNF-*α*, and the inner mechanism is due to abolishment of extracellular signal-regulated kinase 1/2 (ERK1/2) and p38 signaling and then NF-*κ*B inactivation [51, 52]. Similarly, results from Wang et al. and Zhou et al. show that paeonol extenuates contents of ICAM-1, VCAM-1, and E-selection through blocking mitogen-activated protein kinase (MAPKs) and NF-*κ*B cascade, diminishing ECs ability to capture monocytes in the inflammation circumstance [53, 54]. Furthermore, the elevated adhesion of monocytes to ox-LDL-injured vascular ECs is normalized in the presence of paeonol, which is attributed to drug-mediated level promotion of miR-126 delaying the activity of downstream PI3K/Akt/NF-*κ*B axis [55]. Ample literatures have documented that inflammatory factors are cytotoxic that undermine the endothelial barrier and boost the release of proteolytic enzymes, consequently contributing to atherosclerosis onset and destabilization of atheroma plaques [34, 56]. In macrophages, level ascent of IL-1, inducible nitric oxide synthase (iNOS), COX-2, and TNF-*α* elicited by LPS is attenuated after paeonol diminishes activation of Toll-like receptor 4 (TLR4)/NF-*κ*B and ERK1/2 cascade [57, 58]. Moreover, paeonol has been shown to remit inflammation responses via modulating signal flow of other MAPKs such as p38 and c-jun N-terminal kinase (JNK) [59]. According to Choy et al., LPS triggered inflammatory reactions and caused EC apoptosis through stimulating the NADPH/ROS/MAPK cascade and relevant upstream mediator TLR4 and bone morphogenic protein 4 (BMP4), while coadministration with paeonol significantly reversed these events [8]. With microarray analysis, Huang and colleagues proved that paeonol served as an anti-inflammatory agent by repressing actions of signal pathways concerning Toll receptor, interleukin, interferon-*γ*, Janus kinase/signal transducers and activators of transcription, etc. [60]. Apart from directly influencing activities of signal molecules, paeonol has been discovered to prohibit proinflammatory signaling and cytokine generation via affecting specific regulators, as exemplified by paeonol-impeded expression of miR-21 followed by inactivation of Ras/MKK3/6/p38 pathway in ox-LDL-damaged ECs [61]. Additionally, in vivo studies depict that paeonol exerts markedly atheroprotective effects by the way of its proficiency in reducing inflammatory mediators including CRP, TNF-*α*, and IL-1*β* [62].

### 3.4. Improvement of Lipid Profiles and Foam Cell Formation

Compelling evidence indicates that dyslipidemia is one of the crucial activators of atherosclerosis occurrence and progression. Hyperlipidemia perturbs the permeability of vascular wall to promote the proatherogenic sedimentation and oxidation of lipoproteins in the subendothelium. Medications targeting lipid dysbolism have been proved to be effective in controlling atherogenesis [14, 63]. There is evidence establishing that paeonol is able to lower the contents of blood triglyceride (TG), total cholesterol (TC), and low-density lipoprotein cholesterol (LDL-C) and ameliorate atherosclerosis development in rats fed with high-fat diet [64]. Furthermore, in a quail model of atherosclerosis, the decrement of TC, LDL, VLDL, and apolipoprotein (apo) B100 and increment of high-density lipoprotein (HDL), HDL/TC, and apoA1/apoB100 are seen after gavage with paeonol [65]. The lipid-lowering and antiatherogenic effects of paeonol are also identified in a study reported by Qian et al., as assessed by decrease of concentration of TC, TG, and LDL-C and extent of atheroma lesions [66]. In terms of the molecular mechanisms of paeonol-modulated lipid metabolism, Chen and Kang unraveled that paeonol lowered TG level via delaying the de novo synthesis and favoring lipid oxidation through blocking sterol regulatory element-binding protein 1c (SREBP-1c)/fatty acid synthetase (FAS) and SREBP-1c/acetyl CoA carboxylase *α* (ACC*α*) pathway and inducing PPAR-*α*/carnitine palmitoyltransferase I (CPT-1) cascade, respectively. And the decrement of TC and LDL-C was linked to paeonol-evoked depression of 3-hydroxy-3-methylglutaryl-coenzyme A reductase (HMGCR) and ascent of LDL receptor (LDLR), separately [11].

It is widely held that foam cell formation is a hallmark of the early phase of atherosclerosis. Scavenger receptors like CD36, SR-A, and LOX-1 facilitate foam cell formation by internalizing cholesterol, while ATP-binding cassette transporter A1 (ABCA1) and ATP-binding cassette transporter G1 repress macrophage conversion via favoring cholesterol efflux [14, 63]. Recent results have demonstrated that paeonol could activate the liver X receptor *α* (LXR*α*)/ABCA1 pathway to accelerate ox-LDL outflow in macrophages, accompanied by reduction of foam cell formation and pathogenic changes of atherosclerosis [67]. In addition, another study found that paeonol abated macrophages switching into foam cells not only through promoting the efflux of ox-LDL by maintaining stabilization of ABCA1 but also via blocking the cholesterol uptake by abolishing c-Jun-mediated CD36 synthesis and then leading to attenuation of atherosclerosis burden in apoE^−/−^ mice [68]. HDL is reported to be responsible for reverse transport of cholesterol from peripheral organs to the liver, and paeonol is capable of upregulating the circulating level of HDL, implying that increasing HDL might be a mechanism of action for paeonol to expedite cholesterol ejection from macrophages and weaken their conversion [69–71]. Thus, the therapeutic utility of paeonol in atherosclerosis has been at least partly ascribed to regulation of lipid metabolism and suppression of macrophages turn into lipid-laden foam cells.

### 3.5. Suppression of VSMC Growth and Mobilization

Pharmacological efforts with antiproliferatory and antimigratory properties on VSMCs are beneficial for treating atherosclerosis, given that unlimited proliferation and movement of VSMCs within the arterial wall contribute to plaque expansion and vascular narrowing. Once irritated by mitogens, VSMCs in a resting state turn into the synthetic phenotype and begin to proliferate and move from tunica media to intima [72]. Paeonol has been found to restrain phenotype change to suppress VSMC proliferation induced by hyperlipemic serum, suggesting its favorable roles against intima thickening [73]. Furthermore, paeonol decreases platelet-derived growth factor- (PDGF-) triggered VSMC growth by arresting cell cycle at G_0_/G_1_ phase through inactivating mitogenic signal ERK1/2/c-fos [74]. High glucose, one of the predominant contributors to atherosclerosis progression, is capable of promoting EC damage and VSMC growth. It is reported that pretreatment with paeonol markedly reverses high glucose-elicited proliferation of VSMCs in the cell coculture model, and this effect is due to decrease of vascular endothelial growth factor and PDGF release and following blockade of Ras/Raf/ERK1/2 pathway transduction [75]. There is evidence showing that paeonol could decrease the level of blood glucose in hyperglycemic state, hinting that paeonol probably reduces hyperglycemia to indirectly retard VSMC growth [76]. Additionally, TNF-*α*-stimulated enhancement of cellular proliferatory and migratory abilities is restored by paeonol which activates mitochondria-related apoptotic cascade and diminishes extracellular matrix degradation, as explained by increase of Bax and cleaved caspase-9 and caspase-3 and decline of Bcl-2 and matrix metalloprotein (MMP)-2 and MMP-9 [77]. Owing that emerging evidence has determined the implication of autophagy in weakening VSMC growth, Wu and colleagues investigated whether paeonol regulated cellular proliferatory activities via mediating autophagic processes. They discovered that paeonol produced cell cycle arrest in ox-LDL-affected VSMCs and reduced the number of VSMCs in tunica media of apoE^−/−^ mice, both of which were ascribe to the mechanism that paeonol induced enhancement of autophagy via upregulating LC3II expression, p62 degradation, and autophagosome formation through stimulating the AMPK/mammalian target of rapamycin (mTOR) signaling axis [7]. With respect to the roles of paeonol in vascular restenosis, Zhang et al. clarified that local administration of paeonol mitigated early neointimal thickening of graft veins by abrogating mitogenic cytokine-triggered proliferation of VSMCs and apoptosis of ECs, implying the potential application of paeonol for preventing occurrence of in-stent restenosis, a severe complication of angioplasty [78]. According to the above findings, it is apparent that blockade of VSMC proliferation and migration is an important constituent of atheroprotective effects of paeonol.

### 3.6. Repression of Platelet Activity and Thrombosis

Because of circulating hemorheology abnormality or procoagulant material upmodulation in atherosclerotic lesion areas, platelets are extensively activated and recruited to the damaged endothelium, which is an initiation of coagulation cascade, thereby inducing artery thrombosis and vascular occlusion, a life-threatening acute coronary event [79]. Previous studies had deciphered that paeonol and its analogues offered advantageous roles against thrombus formation via directly restraining platelet aggregation and blood coagulation [10, 80]. With improvement of hemorheological parameters, paeonol is considered as a protective agent lessening thrombogenic incidents, by the fact that aberrant whole blood viscosity, plasma viscosity, and fibrinogen participate in coagulation processes [65, 71, 81]. Another antiatherothrombotic effects of paeonol might be associated with the increase of NO and PGI_2_ acting as antagonists of platelet activity while the reduction of ET-1 and TXA_2_ that are agonists of platelet activation and aggregation [82].

## 4. Other Potential Therapeutic Targets

**Figure 3:**
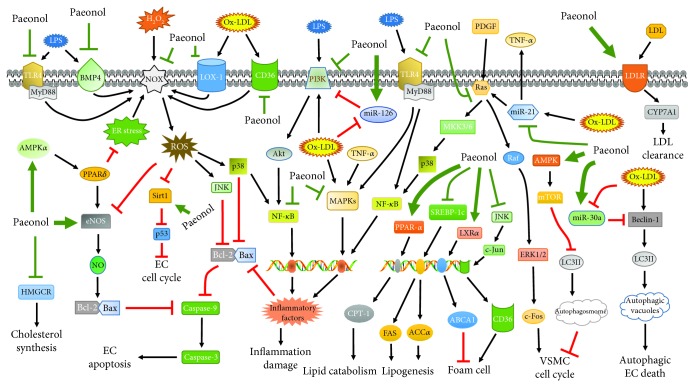
Schematic diagram of molecular mechanism underlying paeonol protects against atherogenesis. Paeonol-induced ROS elimination is associated with inhibition of NOX/ROS pathway. Paeonol mainly suppresses the MAPKs and NF-*κ*B cascade to weaken inflammatory responses and EC apoptosis. In addition, paeonol activates PPAR-*α*/CPT-1 pathway and represses SREBP-1c signaling to accelerate TG catabolism and block TG synthesis, respectively. Then, paeonol weakens foam cell formation by increasing level of reverse transport axis LXR*α*/ABCA1 and reducing activities of JNK signaling involved in CD36 production. Moreover, paeonol mediates autophagic factors and cell cycle-related single molecules to block the VSMC proliferation via the AMPK/mTOR pathway. TLR4: Toll-like receptor 4; MyD88: myeloid differentiation primary response protein 88; AMPK*α*: AMP-activated protein kinase *α*; HMGCR: 3-hydroxy-3-methylglutaryl-coenzyme A reductase; BMP4: bone morphogenic protein 4; PPAR*δ*: peroxisome proliferator-activated receptor *δ*; eNOS: endothelial nitric oxide synthase; NOX: NADPH oxidase; Sirt1: sirtuin 1; LOX-1: lectin-like low-density lipoprotein receptor-1; JNK: c-jun N-terminal kinase; NF-*κ*B: nuclear factor kappa B; PI3K: phosphatidylinositol 3-kinbase; MAPKs: mitogen-activated protein kinases; LPS: lipopolysaccharides; CPT-1: carnitine palmitoyltransferase I; FAS: fatty acid synthetase; ACC*α*: acetyl CoA carboxylase *α*; SREBP-1c: sterol regulatory element-binding protein 1c; MKK: mitogen-activated protein kinase kinase; ABCA1: ATP-binding cassette transporter A1; LXR*α*: liver X receptor *α*; PDGF: platelet-derived growth factor; ERK1/2: extracellular signal-regulated kinase 1/2; mTOR: mammalian target of rapamycin; LC3II: microtubule-associated protein 1 light chain 3 II; LDLR: low-density lipoprotein receptor; CYP7A1: cholesterol 7 alpha-hydroxylase.

Cumulative findings have demonstrated the potential of paeonol in the control of atherosclerotic lesions and held promise for clinical use of paeonol in atherosclerosis treatment [5]. According to the published papers, proatherogenic actions of cells in vascular wall have been effectively inhibited by paeonol, and the molecular mechanisms well investigated have been delineated in Figure 3. Other worthy possible therapeutic targets involved in atheroprotective effects of paeonol are as follows: (1) miRNAs in foam cell formation: given that some miRNAs are related with the processes of macrophages turn into foam cell, such as miR-155 and miR-21, and paeonol is illustrated to be a modulator of this two miRNAs, affecting their expression is likely be a potential target for paeonol to attenuate foam cell formation [61, 83, 84]; (2) autophagy in endothelial impairment: it is recognized that autophagy is required for diverse biological activities including cellular apoptosis, and paeonol offers antiapoptotic effects against ROS-induced myocardial death via abolishing antiautophagic PI3K/Akt/mTOR pathway [42, 85]. Exploring the implication of autophagy in paeonol-mediated ECs protection is rewarding; (3) angiogenesis: paeonol has been viewed as an angiogenesis inhibitor repressing tumor growth and metastasis. As angiogenesis in the atheroma lesions accelerates plaque rupture, it is worth figuring out whether paeonol improves the vulnerability of plaques by regulating angiogenic events in atherosclerotic areas [86, 87]; (4) phenotype switching: paeonol is reported to delay the transformation of VSMCs from quiescent state to proliferatory status, but the mechanisms are poorly defined. Moreover, it is indicated that macrophage polarization from proinflammatory M1 phenotype to anti-inflammatory M2 state is an important contributor to atherosclerosis development. Several molecules regulating the phenotype change have been shown to serve as targets of paeonol, such as miR-21, MAPKs, NF-*κ*B, TNF-*α*, and IL-10 [88, 89]. It can be speculated that improvement of macrophage polarization might be involved in paeonol-reduced atherosclerosis progression; (5) promotion of vasodilation: emerging evidence suggests that paeonol dramatically facilitates arterial dilation through decreasing intercellular calcium content via repressing Ca^2+^ influx and Ca^2+^ release. This vasodilation-promoting feature is promising to explain paeonol-alleviated coronary no-reflow, which is remained to be further elucidated [90, 91]; (6) vascular remodeling: considering that paeonol potently weakens tissue pathological remodeling by abating extracellular matrix production and fibrosis via blocking the transforming growth factor-*β*/Smads cascade, another antiatherogenic target of paeonol is probably linked to restraint of vascular remodeling, a key event favoring expansion of atherosclerotic lesions [3, 92]; and (7) gut microbiota and immunity regulation: evidence has begun to emerge that dysfunction of gut microbiota and autoimmune responses plays encouraging roles in atherogenesis [93, 94]. Seeking effects of paeonol in mediating functions of microbiota and immune cells would provide newly insights into the understanding of antiatherogenic mechanisms of paeonol.

## 5. Conclusion

In summary, considerable research evidence has pointed to the fact that paeonol, a naturally occurring bioactive compound in Cortex Moutan, is a promising therapeutic agent for atherosclerosis management. The antiatherosclerotic roles of paeonol are attributed to its multifactorial actions involving restoring endothelial integrity, repressing oxidative stress, alleviating inflammation, regulating lipid metabolism, inhibiting VSMC proliferation, and ameliorating platelet activation. These pleiotropic pharmacological activities of paeonol suggest great potential of its clinical application in atherosclerosis prevention and treatment. With respect to the undesirable physical characteristics of paeonol, there are reports indicating that several paeonol-loaded carriers have overcome the shortcomings of poor solubility and stability and improving the bioavailability and residence of paeonol in vivo, providing reliable technical support for paeonol in practice. However, the clinical trials monitoring the therapeutic effects of paeonol are scarce in recent years. So, large randomized, controlled, and double-blind trials are urgently needed to evaluate the efficacy and safety of paeonol in atherosclerosis treatment from the perspective of clinical practice.

## Figures and Tables

**Table 1:** The pharmacokinetic parameters of paeonol.

Object	Agent	Route	Dose of paeonol (mg/kg)	*C*_max_ (*μ*g/mL)	*T*_max_ (min)	*T*_1/2_ (min)	MRT (min)	AUC (*μ*g·min/mL)	CL/F (L/kg·min)
Wistar rat plasma	Cortex Moutan	Oral	20	2.69 ± 0.26	19.26 ± 4.4	80.93 ± 16.26	—	172.7 ± 48.86	0.12 ± 0.03
SD rat plasma	Paeonol	Oral	40	3.04 ± 0.61	17.5 ± 5	62.48 ± 17.41	91.25 ± 15.59	334 ± 81.29	0.13 ± 0.03
	Paeonol plus danshensu	Oral	40	0.87 ± 0.08	12.5 ± 5	159.45 ± 56.38	250.85 ± 42.45	186 ± 9.88	0.16 ± 0.04
Wistar rat plasma	Paeonol	Intramuscular	10	0.71 ± 0.13	7.5 ± 2.73	59.85 ± 10.23	77.67 ± 10.48	43.06 ± 6.1	0.24 ± 0.03
SD rat plasma	DA-9805	Oral	58	5.23 ± 3.9	60	90.13 ± 35.97	—	846.82 ± 347.58	—
SD rat plasma	Qingfu Guanjieshu capsule	Oral	70	8.54 ± 1.36	5 ± 0	43.62 ± 3.01	47.97 ± 3.91	265.47 ± 46.71	0.32 ± 0.054
	Qingfu Guanjieshu capsule	Oral	17.75	2.16 ± 0.27	5 ± 0	27.31 ± 1.73	75.5 ± 32	70.78 ± 11.49	0.3 ± 0.06

*C*_max_: the maximum plasma concentration; *T*_max_: the time to reach *C*_max_; *T*_1/2_: half-time of elimination; MRT: mean residence time; AUC: area under the concentration-time curve; CL/F: total clearance; *V*_d_: volume of distribution; DA-9805: a formulation comprising extracts from root cortex of *Paeonia suffruticosa* Andrews, root of Bupleurum falcatum L., and root of Angelica dahurica Benth et Hook; Qingfu Guanjieshu: a formulation containing Caulis Sinomenii, Radix Aconiti Lateralis Preparata, Rhizoma Curcumae Longae, Radix Paeoniae Alba, and Cortex Moutan.
